# The Role of STEAP1 in Prostate Cancer: Implications for Diagnosis and Therapeutic Strategies

**DOI:** 10.3390/biomedicines13040794

**Published:** 2025-03-26

**Authors:** Lingling Zhang, Xinyi Ren, Ran An, Hongchen Song, Yaqi Tian, Xuan Wei, Mingjun Shi, Zhenchang Wang

**Affiliations:** 1 Department of Radiology, Beijing Friendship Hospital, Capital Medical University, Beijing 100050, China; zhanglingling_mail@163.com (L.Z.); renxinyi96@163.com (X.R.); anran8051@163.com (R.A.); tianyqiii@163.com (Y.T.); weixuan315@163.com (X.W.); 2 Department of Urology, Beijing Friendship Hospital, Capital Medical University, Beijing 100050, China; songhcupup@163.com; 3 Institute of Urology, Beijing Municipal Health Commission, Beijing 100054, China

**Keywords:** STEAP1, prostate cancer, biomarker, targeted therapy

## Abstract

Prostate cancer (PCa) is one of the most common malignancies and the second leading cause of cancer-related death in men worldwide. The six-transmembrane epithelial antigen of the prostate 1 (STEAP1) is exceptionally overexpressed in PCa, maintaining high expression even in the castration-resistant prostate cancer (CRPC) stage, making it a promising target for diagnosis and treatment. STEAP1-positive extracellular vesicles and STEAP1-PET imaging are optimistic approaches for the non-invasive detection of different stages of PCa. STEAP1-targeted therapy includes an antibody–drug conjugate (ADC), chimeric antigen receptor T cell (CAR-T), T-cell engager (TCE), and vaccines, which demonstrate valuable therapeutic prospects. This review presents the structure and pathophysiological function of STEAP1, synthesizes cutting-edge advances in STEAP1-targeted molecular imaging and clinical applications, and critically analyzes their translational potential to overcome the limitations of current PCa diagnosis and treatment.

## 1. Introduction

Prostate cancer (PCa) is one of the most common male cancers and is the second leading cause of cancer-related deaths in men worldwide, with 1.47 million new cases and 400,000 deaths in 2022 [1]. Independent risk factors for PCa include age, family history, and genetic susceptibility. Other factors such as smoking, excess weight, diet, and exercise may also play a critical role [2]. Globally, the incidence of PCa is ethnically and geographically diverse. Black men have a higher incidence of PCa and more aggressive disease compared to white or Asian men [3]. The incidence in developed countries is about three times higher than that of developing countries (35.5 and 12.6 per 100,000, respectively), despite the difference in mortality not being significant, which may reflect the importance of early screening. Such screening includes a digital rectal examination and serum prostate-specific antigen (PSA) testing. In developed countries, widespread PSA screening has led to earlier detection and artificially inflated incidence rates, while developing countries face systemic underdiagnosis due to limited healthcare infrastructure and a lack of routine screening programs. The burden of PCa in developing countries is anticipated to grow substantially in future decades, driven primarily by aging populations and increasing life expectancy [4]. Multiparametric magnetic resonance imaging (MRI) examination has been commonly used for suspected abnormal PSA levels. In some cases, a more specific test—prostate-specific membrane antigen (PSMA) positron emission tomography (PET)—is also essential. However, given the heterogeneous expression of PSMA in a subset of PCa and the limited specificity of PSA for aggressive disease, there is an imperative need for biomarkers with dual diagnostic and therapeutic potential, such as the six-transmembrane epithelial antigen of the prostate 1 (STEAP1), which is overexpressed in PCa [5,6,7]. Histopathologic biopsy diagnosis is still the gold standard, with the assistance of MRI and transrectal ultrasound for localization [8].

The clinical management of localized early-stage PCa depends on the PSA level, clinical TNM staging, Gleason scores, and a combination of the patient’s age, preferences, and comorbidities. Patients with low- and intermediate-risk tumors tend to be treated with radical prostatectomy or radiotherapy, and even active surveillance for selected cases [9]. High-risk tumors require systematic, sustained androgen deprivation therapy (ADT).

Since the development of PCa is inextricably linked to the androgen receptor (AR), ADT therapy is generally effective for treatment-naive tumors; this is also called the hormone-sensitive prostate cancer (HSPC) stage, for which AR signaling inhibitors (ARSIs) with AR antagonism (such as enzalutamide) or androgen synthesis inhibition (such as abiraterone acetate), as well as gonadotropin-releasing hormone (GnRH) agonists and antagonists (such as degarelix), are often chosen. However, over time, a proportion of PCas inevitably progress to castration-resistant prostate cancer (CRPC). As a result, treatments other than ADT, such as chemotherapeutic agents (such as docetaxel), poly ADP–ribose polymerase (PARP) inhibitors (such as olaparib), immune checkpoint inhibitors, targeted therapies, and combination regimens, are highly recommended [10].

Targeted therapy is promising given its precision in hitting the lesion with fewer side effects. ^177^Lu-PSMA-617 (Pluvicto^TM^), which targets PSMA-positive PCa, is a successful example, which was approved by the FDA in early 2022 [11]. In addition to PSMA, emerging targets such as STEAP1—a transmembrane protein first discovered in PCa—hold promise [12]. Although STEAP1 is overexpressed in multiple cancers, its expression is exceptionally high in PCa, combined with its functional linkage to disease progression, making it an optimal target for detection and treatment. As a result, studies on STEAP1 have gradually increased in recent years, especially in the field of PCa. Our review focuses on STEAP1 and summarizes its functions and applications in terms of structure, pathology, diagnosis, and treatment.

## 2. Structure and Oncogenic Properties of STEAP1

STEAP1 belongs to the STEAP protein family, and its coding gene, composed of 339 amino acids, is located on chromosome 7q21.13. It has six transmembrane helical domains, two intracellular rings, and three extracellular rings, with intracellular hydrophilic C- and N-terminals at each end [13]. The C-terminal transmembrane domain is homologous to the yeast ferric reductase family of b-type cytochrome metalloreductases. The N-terminal cytoplasmic domain does not exhibit the same characteristics as other members of the STEAP protein family, that is, a structure similar to the F_420_H_2_:NADP^+^ oxidoreductase (FNO) domain [14,15]. Because the FNO-like domain is involved in transmembrane electron transport and the reduction of Fe^3+^ and Cu^2+^, STEAP1 was initially thought to have no metal reductase activity [16,17]. However, STEAP1 was partially co-localized with transferrin and transferrin receptor 1, suggesting that it may be involved in iron metabolism [14]. Kim K et al. found that STEAP1 can transfer electrons through B-heme and reduce metal ion complexes [18]. Further studies showed that STEAP1 can fuse to the NADPH binding region of STEAP4 and participate in iron metabolism by forming heterotrimers [13].

**Figure 1:**
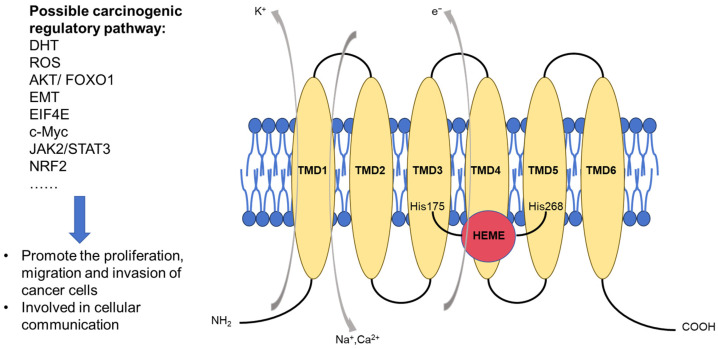
STEAP1 protein structure, biological function, and possible carcinogenic regulatory pathway. TMD: transmembrane domain; DHT: dihydrotestosterone; ROS: reactive oxygen species; AKT/FOXO1: protein kinase B/forkhead box protein O1; EMT: epithelial–mesenchymal transition; EIF4E: eukaryotic initiation factor 4E; JAK2/STAT3: Janus kinase 2/signal transducer and activator of transcription 3; NRF2: nuclear erythroid 2-related factor.

STEAP1 is widely overexpressed in various cancers, such as bladder cancer and Ewing’s sarcoma, but is exceptionally high in PCa [12]. In PCa cell lines, LNCAP, C42B, VCAP, and 22RV1 demonstrate high STEAP1 expression, while PC3 shows low expression [19]. In different human PCa models, STEAP1 can be regulated by AR [20] and may impact dihydrotestosterone activity [6,21]. It has been shown that inhibiting the expression of STEAP1 in tumor cells may reduce proliferation and invasion in vitro, as well as inducing cell apoptosis and inhibiting metastasis in vivo. In addition, STEAP1 may have an influence on tumor behavior through many different signaling pathways, such as the epithelial–mesenchymal transition (EMT) regulated by the JAK/STAT pathway in lung adenocarcinoma [22], c-Myc-mediated proliferation and cell cycle arrest in hepatocellular carcinoma [23], AKT/FOXO1 and EIF4E signaling-mediated invasion in gastric cancer [24,25], and NRF2 pathway-provoked cell apoptosis in colorectal cancer [26]. Interestingly, STEAP1 is also involved in cancer cell communication. Challita-Eid PM et al. blocked STEAP1 expression with monoclonal antibodies and found intercellular communication was inhibited in a dose-dependent manner [27]. Furthermore, Yamamoto T et al. demonstrated that STEAP1 is involved in tumor growth through a small-molecule solute’s intercellular communication with neighboring tumor-associated stromal cells, rather than through direct communication through gap junctions [28]. The structure, biological function, and possible carcinogenic regulatory pathways of the STEAP1 protein are shown in Figure 1.

## 3. STEAP1 as a Promising Biomarker for PCa Diagnosis

Serum PSA is an important tool for early PCa screening, but it lacks specificity, since elevated PSA levels can also be seen in non-malignant diseases such as benign prostatic hyperplasia (BPH) or prostatitis. The sensitivity and specificity of PSA in identifying PCa were 0.93 (95% CI 0.88–0.96) and 0.20 (95% CI 0.12–0.33), respectively [5]. In contrast, STEAP1 was moderately or highly expressed in the majority of PCa patients (88.89%, 56/63), while low or no expression was demonstrated in most BPH patients (95.12%, 39/41), thus confirming the power of STEAP1 in distinguishing malignant lesions from BPH, with 100% (95% CI 94.9–100.0) sensitivity and 95.1% (95% CI 83.5–99.4) specificity [29]. In PCa, STEAP1 overexpression correlates with elevated Gleason scores. It serves as an independent predictor of biochemical recurrence (BCR) and is associated with reduced BCR-free survival [30]. Beyond PCa, STEAP1 dysregulation predicts an unfavorable prognosis across multiple malignancies. In Ewing family tumors, elevated STEAP1 expression correlates with diminished metastasis-free survival (MFS), defined as the interval from treatment initiation to the first occurrence of localized lymph node or distant organ metastasis. Similarly, STEAP1 overexpression is linked to poorer overall survival (OS) in colorectal cancer, diffuse large B-cell lymphoma, acute myeloid leukemia, and multiple myeloma [31]. Notably, patients with high expressions of both STEAP1 and STEAP2, or of both STEAP1 and STEAP4, had better chances of survival compared to those with only STEAP1 overexpression [32]. Consequently, STEAP1 may be a promising biomarker in PCa diagnosis and prognosis.

It has been reported that STEAP1 is enriched in extracellular vesicles (EVs). STEAP1-positive EVs were elevated in PCa and can be used as circulating biomarkers for diagnosis (sensitivity: 76.79%; specificity: 100%) but not for risk stratification [33]. Palmitoylation is crucial for sorting proteins into EVs. The proportion of STEAP1 in EVs was reduced when palmitoylation was inhibited, implying a specific post-translation modification is necessary for normal function. Moreover, Minciacchi VR et al. speculated that STEAP1-positive EVs with different sizes or EpCAM statuses might have different prognostic effects [34]. Recently, Carvalho M et al. developed an electrochemical biosensor for detecting STEAP1 based on a molecularly imprinted polymer and a nanocomposite of dendritic platinum nanoparticles aminated with carbon nanotubes. Their sensor displayed high selectivity, high sensitivity, and good repeatability [35].

The PSMA PET imaging agent ^68^Ga-PSMA-11 has been approved by the FDA and has been widely used in PCa diagnosis and staging. However, as PSMA was closely regulated by AR, its expression in PCa was heterogeneous within treated samples, leading to inaccuracies in molecular imaging and cancer surveillance [36,37]. STEAP1 persistence in CRPC supports its application as an imaging biomarker across PCa stages [6]. Indeed, preclinical models have demonstrated that ^89^Zr-DFO-MSTP2109A, derived from a fully humanized monoclonal STEAP1 antibody, cannot only detect PCa with high STEAP1 expression but also reflect treatment effectiveness by measuring the expression in these models [20]. In addition, the authors found a high contrast of ^89^Zr-DFO-MSTP2109A localization in both bone and soft tissue metastases, where the median standardized uptake values (SUVs) were 20.6 and 16.8, respectively. A bone biopsy showed that 86% of histologically positive lesions were radiographically true positive. This was superior to the anti-PSMA antibody-based imaging agent ^89^Zr-IAB2M (bone mean SUVmax: 13.8, soft tissue mean SUVmax: 7) [38]. Moreover, the imaging agent accumulates less in the liver tissue, meaning liver metastases can be better displayed [39,40]. All these results support the idea that STEAP1 might be a better biomarker than PSMA in identifying metastatic PCa lesions. A comparative evaluation of PCa biomarkers (PSA, PSMA, and STEAP1) is provided in Table 1. Yuan Y et al. also showed that STEAP1-conjugated SonoVue microvesicles not only had a significantly higher ultrasonic signal intensity than ordinary SonoVue microvesicles but also significantly improved the peak intensity in the LNCaP xenograft model (13.656 ± 3.12%) compared to the PC3 xenograft model (3.986 ± 2.24%). This is consistent with the STEAP1 expression in these cell lines [41]. Sun J et al. developed STEAP1-targeted extracellular vesicles (AS-EVs) encapsulating near-infrared fluorescent dye S0456 for fluorescence-guided surgery (FGS). Preclinical validation demonstrated rapid blood clearance (t1/2 = 4.29 h), sustained tumor retention (>72 h, tumor background ratio 3:1), and superior specificity over controls, enabling FGS in xenograft models to reduce positive surgical margins and enhance postoperative survival (*p* = 0.0342) [42]. Together, molecular imaging targeting STEAP1 can help in disease diagnosis and monitoring and the selection of antigen-directed therapies.

## 4. STEAP1 as a Potential Therapeutic Target

**Figure 2:**
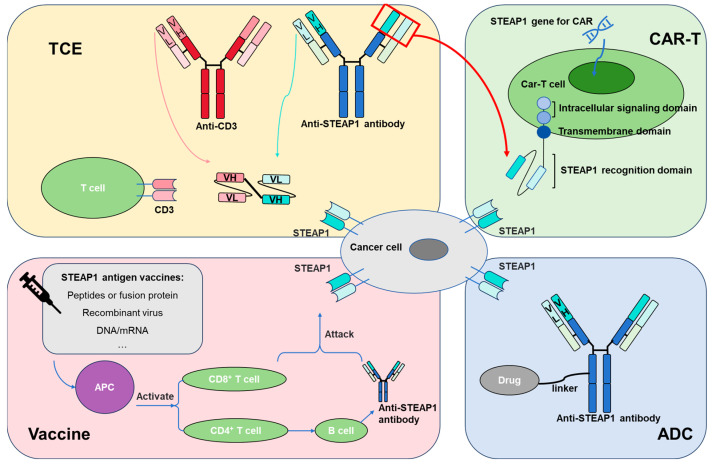
Schematic diagram of the mechanism of action between STEAP1-expressing PCa cells and ADC, CAR-T, TCE, and vaccines. PCa: prostate cancer; STEAP1: six-transmembrane epithelial antigen of the prostate 1; ADC: antibody–drug conjugate; CAR-T: chimeric antigen receptor T cell; TCE: T-cell engager; VH: variable heavy chain; VL: variable light chain; APC: antigen-presenting cell.

The fact that STEAP1 is highly expressed in PCa and shows low or no expression in normal tissues makes it an ideal therapeutic target. The following sections will introduce four targeted therapy methods: antibody–drug conjugate (ADC), chimeric antigen receptor T cell (CAR-T), T-cell engager (TCE), and vaccines. The mechanism of action between STEAP1-expressing PCa cells and these therapies is shown in Figure 2. We also summarized clinical and preclinical evidence targeting STEAP1 in Table 2.

### 4.1. ADC

An ADC connects a monoclonal antibody that specifically recognizes a tumor cell surface antigen with a cytotoxic drug via a chemical linker; subsequently, it enters the cancer cell through endocytosis. With the participation of lysosomes, linkers break due to a low pH or specific enzymes, releasing cytotoxic drugs that kill cancer cells by interfering with DNA replication or microtubule function. Through the targeting ability and powerful lethality of cytotoxic drugs, ADCs achieve precise attacks on cancer cells while minimizing damage to normal cells. In 2019, Danila DC et al. conducted a phase I study on DSTP3086S in an mCRPC population. DSTP3086S is an ADC drug that consists of a humanized monoclonal STEAP1 antibody linked to monomethyl auristatin E (MMAE) via a protease labile linker. A total of 84 patients with PCa were enrolled in the study, of which 7 received weekly treatment and 77 received treatment once every 3 weeks. Of the 84 patients, 3 had dose-limiting toxicity events, which were defined as grade ≥ 3 nonhematologic or grade ≥ 4 hematologic toxicities during the first treatment cycle. In the once-every-3-week dose group, 34% of treatment-related adverse events (TRAEs) had a grade of three or higher, with fatigue and peripheral neuropathy being most common. About 29% (18/62) of patients achieved a PSA response, 59% (16/27) showed good circulating tumor cell conversions, and 4% (2/46) saw a partial response according to response evaluation criteria in solid tumors (RECIST) [53].

ADRX-0405 is a next-generation ADC consisting of a humanized IgG1 antibody targeting STEAP1. The i-Conjugation^®^ technology platform, which enhances payload delivery with protease-cleavable linkers and stable conjugation chemistry, ensures ADC stability, with a topoisomerase inhibitor payload with a drug-to-antibody ratio of eight. Preclinical studies focusing on ADRX-0405 have demonstrated favorable pharmacokinetics, a good safety profile, and significant efficacy across multiple tumor models. Researchers are currently recruiting for the first in-human phase Ia/b clinical trial of ADRX-0405 in an open-label dose escalation and dose expansion study (NCT06710379). DXC008 is an ADC drug that targets STEAP1 with a tubulysin B analog payload. Not only does the drug have a high affinity for STEAP1 but also a moderate affinity for PSMA. DXC008 has demonstrated high levels of activity against several prostate tumor cells in vitro and has shown very good persistent antitumor response in vivo in xenograft models. At present, applications are being submitted to the National Medical Products Administration (NMPA) of China (CXSL2400868) for clinical trials with DXC008.

### 4.2. CAR-T

Although CAR-T is more extensively applied in hematological diseases, it is also rapidly developed in solid tumors [65]. For example, anti-claudin18.2 CAR-T showed a good anti-gastric cancer effect [66]. Jin Y et al. designed a CAR-T in which Oslo1 scFv was derived from variable heavy- and light-chain sequences identified by the American Type Culture Collection, PTA-5803 hybridoma. The retroviral vector SFG was labeled with the RQR8 gene and the CD8a spacer, transmembrane domain, and 4-1BB co-stimulatory domain were introduced. Both CD4^+^ and CD8^+^ T cells acquired the desired STEAP1-specific activity, including the production of multiple cytokines, proliferation, and the killing of target cells. It was encouraging that the efficacy of anti-STEAP1 CAR-T in vitro was comparable to that of anti-CD19 CAR-T. Anti-STEAP1 CAR-T shows antitumor activity in both subcutaneous and metastatic xenograft mouse models of PCa [56].

Anti-STEAP1-BBζ CAR-T is based on the pCCL-c-MNDU3-X lentiviral skeleton, where CAR expression in T cells is driven by an internal MNDU3 promoter. Bhatia V et al. constructed anti-STEAP1-BBζ CAR-T cells that exhibited obvious antitumor activity in models of metastatic PCa both in human and mouse studies. Consistent with previous studies, T-cell activation and cytolytic activity were observed, even in cases with low STEAP1 antigen density, which enhanced antitumor efficacy. No off-target tumor toxicity was found in subsequent humanized STEAP1 mouse models. Importantly, using CBD-IL-12 in combination with CAR-T overcomes the immunosuppressive microenvironment of PCa [6]. Currently, researchers are recruiting for a phase I/II trial (NCT06236139) testing the safety and effectiveness of anti-STEAP1 CAR-T combined with enzalutamide in treating patients with mCRPC, expecting to enroll 48 patients.

### 4.3. TCE

TCE is a targeted immunotherapeutic strategy that induces T-cell activation and mediates cytotoxic T cells in order to kill tumor cells via the bispecific binding of T-cell surface molecules to tumor-associated antigens on target cells [67,68]. AMG 509 (Xaluritamig) is an XmAb 2+1 T-cell contactor targeting STEAP1; it consists of two fragment antigen-binding domains that bind STEAP1, one anti-CD3 single-chain fragment variable domain, and one fragment crystallizable immunoglobulin domain, and specifically mediates T-cell-dependent cytotoxicity in PCa and Ewing’s sarcoma cells expressing high levels of STEAP1 [19,69]. In a phase I trial (NCT04221542) of 97 mCRPC patients treated with AMG 509, 55% of patients developed TRAEs greater than grade 3, with cytokine release syndrome (CRS) (72%), fatigue (45%), myalgia (34%), and fever (32%) being most common. Of the 67 RECIST-evaluable patients, 16 (24%) showed a complete response, while PSA responses were found in 43 (49%) patients of the 87 assessable. In the high-dose cohort, the objective RECIST and PSA response rates reached 41% and 59%, respectively, and 25% (13/52) of patients continued treatment for more than six months. Trials are currently underway to explore the safety, pharmacokinetics, and efficacy of the maximum tolerated dose [70]. The next part of this study is now recruiting in order to evaluate the efficacy of AMG 509 monotherapy in chemotherapy-naive patients who have received one NHT treatment, as well as the combination of AMG 509 with abiraterone or enzalutamide in CRPC patients who have previously received one non-hormonal therapy and up to one paclitaxel treatment.

### 4.4. Vaccine

Therapeutic cancer vaccines now show signs of efficacy and potential in delaying tumor growth in mice, improving the tumor microenvironment, significantly prolonging the OS rate of tumor-bearing mice, inhibiting tumor cell growth, and activating cytokines in human cell lines [71]. STEAP1-derived immunogenic peptides activate cytotoxic CD8^+^ T cells through the presentation of MHC-I class cells. There were already several STEAP1 vaccines with enhanced immunogenicity: STEAP_86–94_, STEAP_262–270_, STEAP_292–300_, STEAP_186–193_, STEAP_102–116_, and STEAP_192–206_ [62,72,73,74]. These include fusion protein, recombinant virus, and DNA/mRNA vaccines [62,63,64]. The vaccination method also reportedly plays a role in effectiveness. For example, intravenous enhancers increased the in vivo killing compared to intramuscular enhancers [75]. Importantly, successive vaccinations with different antigen-presenting systems encoding the same antigen, such as recombinant DNA and viruses-encoding STEAP1, tend to have stronger immune effects [76,77]. Remarkably, the combined use of STEAP1 antigen vaccines has a stronger antitumor effect than a single vaccine [77].

Novel vaccines containing coding sequences for multiple PCa-expressing antigens also showed significant antitumor activity [64,75]. Thorne AH et al. reported that, when added to vaccine preparations, adjuvants composed of cytokines, chemokines, and T cell co-stimulants can simulate the effects, enhance T-cell response intensity, and display good auxiliary effects [78]. A phase I/IIa study demonstrated that CV9103, a self-adjuvant mRNA PCa vaccine containing the coding antigens PSA, PSCA, PSMA, and STEAP1, was well-tolerated and immunogenic in patients with CRPC. The most frequent treatment-related side effects were injection site erythema and reactions, fatigue, pyrexia, chills, and influenza-like illness. Of the 33 evaluable patients who received CV9103, 78.8% presented an immune response and 45.5% responded to multiple vaccination antigens. However, when introducing more antigen-encoding mRNA, such as prostatic acid phosphatase and Mucin 1 to create CV9104 (based on the CV9103 backbone), no further survival benefit was observed in the I/IIB trial [61,79]. Cappuccini F’s study indicates that the ChAdOx1-MVA vaccination regimen targeting STEAP1, combined with anti-PD-1 treatment, significantly improved the survival rate of mice [80]. Together, vaccines targeting STEAP1 have high clinical therapeutic potential.

Sipuleucel-T, the first FDA-approved vaccine, is an autologous active cellular immunotherapy used for patients with asymptomatic or minimally symptomatic mCRPC. It was developed from autologous dendritic cells (DCs) loaded with an engineered fusion protein of prostatic acid phosphatase (PAP) and granulocyte–macrophage colony-stimulating factor (GM-CSF). This therapeutic vaccine prolongs OS in mCRPC patients, albeit with minimal improvement in progression-free survival (PFS). TRAEs were reported in nearly all recipients, which were predominantly mild to moderate; these TRAEs, such as chills, fatigue, and fever, were common in more than 30% of patients. Other vaccine candidates remain in preclinical or clinical development [81,82].

### 4.5. Limitations of Targeted Therapy

Off-target effects refer to the effects of a drug on non-malignant tissue expressing the target antigen. Despite the absence or low expression of STEAP1 in normal tissues, toxicity from off-target effects may still cause harm to normal tissues [83,84,85,86]. The tumor microenvironment also plays an important role in the practical application of drugs. In particular, unlike hematological malignancies, PCa is a “cold” tumor with an immunosuppressive tumor microenvironment, which may affect its therapeutic effectiveness [87,88]. In addition, different targeted therapies also have specific side effects. Currently, there are seven FDA-approved CAR-T drugs, all of which are used to treat hematological disorders. The characteristic toxicity of CAR-T cells includes CRS, immune effector cell-associated neurotoxicity syndrome (ICANS), and immune effector cell-associated hemophagocytic lymphohistiocytosis (HLH)-like syndrome (IEC-HS) [89]. Of the patients with nonrecurrent death, 11% died from CAR-T-cell-specific side effects up to March 2024 [90]. Similar to CAR-T, CRS and ICANS are common TRAEs of TCE. Compared to CAR-T cells, TCE is responsible for less frequently severe CRS or ICANS. The other common reactions and TRAEs associated with TCE include infections, tumor flare reactions, and cytopenia [91]. As a coupling drug commonly used in ADCs, MMAE has the typical side effect of tubulin binders, inducing peripheral neuropathy. Myeloid toxicity is a common complication of most cytotoxic chemotherapeutic drugs. In addition, there are some unexpected toxicities, such as pulmonary and ocular, in ADC clinical trials. ADC drug development often ends when the maximum toxicity is reached without a satisfactory therapeutic effect [85]. Despite the rapid development of cancer vaccine research, immunogenicity and stability are still issues that need continuous attention and improvement [92]. Innate immunity should be appropriately activated to initiate an adaptive immune response while avoiding the toxic overactivation that inhibits antigen protein expression and immune response [93]. Cancer vaccines are often associated with mild TRAEs, such as infusion reactions characterized by flu-like symptoms and myalgia. Targeted antigens, adjuvant types, different platforms, immunogenicity, and other factors can affect TRAEs [94].

## 5. Conclusions

There is still an urgent need for the early detection of PCa, a disease that represents challenging malignancies regarding treatment options. STEAP1, a PCa membrane protein, not only demonstrates potential as a sensitive biomarker for cancer detection through plasma EVs or molecular imaging examinations but also demonstrates promising therapeutic utility via direct or indirect targeting. It is believed that more research on the mechanism of STEAP1, and innovative therapies targeting it, will continue to emerge, bringing more personalized and effective treatment options for PCa patients.

## Figures and Tables

**Table 1:** Comparative analysis of prostate cancer biomarkers: PSA, PSMA, and STEAP1.

Biomarker	Key Characteristics	Diagnostic Utility	Therapeutic Utility	Limitations
PSA	Glycoprotein elevated in PCa Low tissue specificity	Serum screening Disease surveillance [43]	None (prognostic marker only)	False positives (BPH/inflammation) Limited prognostic value in advanced stages [5]
PSMA	Transmembrane protein Overexpressed in advanced PCa	Imaging: PSMA-PET (high sensitivity) [44], PSMA-SPECT [45], PSMA-FLU [46], PSMA-US [47], PSMA-PA [48], multimodal imaging [49,50]	A relatively mature targeted therapy program [51]	Heterogeneous expression across metastases [6] Normal tissue uptake (salivary glands) [52]
STEAP1	6-transmembrane protein Upregulated in advanced PCa Involved in intercellular communication [27]	EV-based liquid biopsy [33] Low heterogeneity Imaging: STEAP1-PET [20], STEAP1 US [41], STEAP1-FLU [42]	Potential of targeted therapy	Limited clinical validation Similar to PSMA, it is regulated by AR [20]

Prostate cancer: PCa; six-transmembrane epithelial antigen of the prostate 1: STEAP1; prostate-specific antigen: PSA; prostate-specific membrane antigen: PSMA; positron emission tomography: PET; single photon emission computed tomography: SPECT; fluorescence: FLU; ultrasound: US; photoacoustic: PA; benign prostatic hyperplasia: BPH; androgen receptor: AR.

**Table 2:** Therapeutic agents that target STEAP1.

Drug Class	Drug Name	Company or Author	Report Date/Launch Date	Status	Cancer	Study/Citation
ADC	DSTP3086S	Danila DC	2019	Phase I Completed	mCRPC	NCT01283373 [53]
ADC	ADRX-0405	Adcentrx Therapeutics	2024	Phase Ia/b Recruiting	Advanced Solid Tumors	NCT06710379 [54]
ADC	DXC008	DAC Biotechnology	2024	Phase I Recruiting	PCa	CXSL2400868 [55]
CAR-T	CAR-T with Oslo1 scFv and 4-1BB co-stimulatory domain	Jin Y	2022	Preclinical	Mouse PCa	[56]
CAR-T	anti-STEAP1-BBζ CAR-T +Enzalutamide	Bhatia V	2023	Phase I/II Recruiting	mCRPC	NCT06236139 [6]
TCE	AMG 509 (Xaluritamig)	Amgen	2020	Phase I Recruiting	mCRPC	NCT04221542 [57]
TCE	AMG 509 (Xaluritamig)	Amgen	2024	Phase I Recruiting	nmCSPC	NCT06555796 [58]
TCE	AMG 509 (Xaluritamig)	Amgen	2024	Phase I Recruiting	Localized Pca	NCT06613100 [59]
TCE	BC261	Lin TY	2021	Preclinical	Mouse EFT and Pca	[60]
mRNA vaccine	CV9104	Kübler H	2015	Phase I/IIB Completed	mCRPC	NCT01817738 [61]
Fusion protein vaccine	Ag85B-3 × STEAP1_186–193_	Guo L	2021	Preclinical	PCa	[62]
Fusion protein vaccine	HSP65 and STEAP1_186–193_	Chen X	2019	Preclinical	Mouse PCa and Melanoma	[63]
DNA vaccine	PCaA-SEV	Bordoloi D	2021	Preclinical	Mouse PCa	[64]
